# Identification of Flavonoids from *Scutellaria barbata* D. Don as Inhibitors of HIV-1 and Cathepsin L Proteases and Their Structure–Activity Relationships

**DOI:** 10.3390/molecules28114476

**Published:** 2023-05-31

**Authors:** Ting-Ting Tang, Su-Mei Li, Bo-Wen Pan, Jun-Wei Xiao, Yu-Xin Pang, Shou-Xia Xie, Ying Zhou, Jian Yang, Ying Wei

**Affiliations:** 1 College of Pharmacy, Guizhou University of Traditional Chinese Medicine, Guiyang 550025, China; tangingtt@163.com (T.-T.T.); pandawater@163.com (B.-W.P.); sethverlo77@hotmail.com (J.-W.X.); pyxmarx@126.com (Y.-X.P.); 2 Department of Pharmacology, Shenzhen People’s Hospital (The Second Clinical Medical College, Jinan University, The First Affiliated Hospital, Southern University of Science and Technology), Shenzhen 518020, China; li.sumei@szhospital.com (S.-M.L.); szshouxia@163.com (S.-X.X.); 3 College of Pharmacy and Nutrition, University of Saskatchewan, 107 Wiggins Road, Saskatoon, SK S7N 5E5, Canada; jian.yang@usask.ca

**Keywords:** *Scutellaria barbata* D. Don, flavonoids, HIV-1 protease, cathepsin L protease, structure–activity relationships

## Abstract

*Scutellaria barbata* D. Don (**SB**, Chinese: Ban Zhi Lian), a well-known medicinal plant used in traditional Chinese medicine, is rich in flavonoids. It possesses antitumor, anti-inflammatory, and antiviral activities. In this study, we evaluated the inhibitory activities of **SB** extracts and its active components against HIV-1 protease (HIV-1 PR) and SARS-CoV2 viral cathepsin L protease (Cat L PR). UPLC/HRMS was used to identify and quantify the major active flavonoids in different **SB** extracts, and fluorescence resonance energy transfer (FRET) assays were used to determine HIV-1 PR and Cat L PR inhibitions and identify structure–activity relationships. Molecular docking was also performed, to explore the diversification in bonding patterns of the active flavonoids upon binding to the two PRs. Three **SB** extracts (SBW, SB30, and SB60) and nine flavonoids inhibited HIV-1 PR with an IC_50_ range from 0.006 to 0.83 mg/mL. Six of the flavonoids showed 10~37.6% inhibition of Cat L PR at a concentration of 0.1 mg/mL. The results showed that the introduction of the 4′-hydroxyl and 6-hydroxyl/methoxy groups was essential in the 5,6,7-trihydroxyl and 5,7,4′-trihydroxyl flavones, respectively, to enhance their dual anti-PR activities. Hence, the 5,6,7,4′-tetrahydroxyl flavone scutellarein (HIV-1 PR, IC_50_ = 0.068 mg/mL; Cat L PR, IC_50_ = 0.43 mg/mL) may serve as a lead compound to develop more effective dual protease inhibitors. The 5,7,3′,4′-tetrahydroxyl flavone luteolin also showed a potent and selective inhibition of HIV-1 PR (IC_50_ = 0.039 mg/mL).

## 1. Introduction

Globally, 766 million confirmed cases of coronavirus disease 2019 (COVID-19), including 6.93 million deaths, have been reported to the World Health Organization (WHO) [1]. Approximately 10–20% of people infected with severe acute respiratory syndrome coronavirus 2 (SARS-CoV-2) develop long COVID-19, with sequelae such as fatigue, shortness of breath, persistent cough, depression and anxiety, brain fog, myocardial inflammation, and myocardial infarction. In particular, cardiovascular health and mortality are emerging as a new epidemic, substantially changing the lives of millions of people globally [2]. To date, 1.33 trillion COVID-19 vaccines have been administered [1]. However, SARS-CoV-2 mutates quickly and is highly infectious, disparities in vaccinations exist, and effective treatments for long COVID-19 are lacking. Current challenges include the development of more effective vaccines and drugs against COVID-19 [2,3]. During experiments of antiviral drug discovery, two types of anti-SARS-CoV-2 agents, virus-protein targeted agents (blocked virus life cycle) and host protein targeted agents (involved in the viral life cycle), need to be developed to tackle COVID-19 and long COVID-19 [4]. Since both SARS-CoV-2 and HIV are RNA viruses relying on proteases for maturation, HIV-1 protease (HIV-1 PR), an aspartic protease, and cathepsin L protease (Cat L PR), a cysteine protease, have been shown to play a vital role in the lifecycle of HIV and SARS-CoV-2, and are considered key antiviral drug targets [5,6]. 

In spite of SARS-CoV-2 Cat L PR being a different class of protease (cellular protease) [4], several HIV-1 PR inhibitors have exhibited potent activities towards Cat L PR [7]. Combinations with other therapeutics are currently under clinical trial for the treatment of COVID-19 [7]. Furthermore, the US Food and Drug Administration (FDA) advisory committee has recommended full approval of Paxlovid (Nirmatrelvir/Ritonavir) for treatment of COVID-19 [8]. Although Nirmatrelvir is a substrate of cytochrome P450 (CYP) 3A4, co-administration of Ritonavir can significantly reduce the CYP3A4 metabolism of Nirmatrelvir and result in a high serum level [8]. Other promising drug combinations including Nelfinavir/Cepharanthine and Lopinavir/Ritonavir have also been investigated for COVID-19 treatment [9,10]. 

Over the past decade, seven Cat L inhibitors have been developed as anti-coronavirus agents, with K111777 and oxocarbazate being the most promising candidates [6]. Furthermore, recent studies have shown that ten FDA-approved drugs exhibited Cat L inhibitory activity [6]. These drugs could be repositioned for COVID-19 treatment, especially for patients in the early stages of infection or who are asymptomatic. Cat L inhibitors can block viral entry on the surface of host cells, without affecting the adaptive immunity [6]. However, some serious adverse reactions may arise from drug–drug interactions [9]. Owing to these drug–drug interactions and rapid development of viral resistance, a continued search for HIV-1 PR or/and Cat L PRs inhibitors is critical for the prevention and treatment of SARS-CoV-2 and other viral infections [6,8]. 

Plant-derived natural products remain a rich source of therapeutic agents for human illnesses [11]. Some natural products and their derivatives have exhibited antiviral activities and are under clinical trial as potential therapeutic agents [12]. *Scutellaria barbata* D. Don (**SB**, Chinese: Ban Zhi Lian) is a well-known traditional medicinal plant widely distributed in Japan, Korea, and China. It has been used in a wide range of applications in folk medicine, including treatment of snakebites, tumors, hepatitis, sore throat, pulmonary abscess, and hemoptysis. In the 2020 edition of the Chinese Pharmacopoeia (volume one), **SB** (tablet or extract) is listed for medicinal use in treating pneumonia, bronchitis, pharyngitis, and pulmonary abscess [13,14]. More than 200 components, predominately flavonoids and neoclerodane diterpenoids, have been isolated and characterized from methanol or ethanol extracts of **SB** whole grass or aerial parts [15]. The potential therapeutic effects of **SB** extracts and major components have been extensively studied. Huang et al. reported that **SB** aqueous extracts and six major flavonoids prevented SARS-CoV-2 infection via inhibition of Mpro and TMPRSS2 proteases [16]. Zhou et al. screened nine flavonoids obtained from **SB** for their anti-HBV activity [17]. Some flavones in **SB** were shown to prevent parainfluenza viral infection [18]. Moreover, anti-cardiovascular disease activity was observed for **SB** extracts and some flavones during both in vivo and in vitro studies [19]. However, the inhibitory effects of **SB** extracts and its flavonoids against HIV-1 and Cat L PRs have not been investigated. Thus, in the current study, we evaluated the anti-HIV-1 PR and anti-Cat L PR activities of four extracts and nine flavonoids from **SB**.

## 2. Results and Discussion

### 2.1. Inhibition of HIV-1, Cat L, and Renin PRs by ***SB*** Extracts

**SB** extract was prepared from the whole plant according to a previously described method [20]. Using a high-throughput screening approach [20,21], the safety of four **SB** extracts (SBW, SB30, SB60, and SB85: prepared from water or MeOH-H_2_O) was initially evaluated using renin protease (Table 1) (Please refer to the Supplementary Materials for details). SBW and SB30 had anti-human renin PR activities with IC_50_ values of 0.59 and 0.70 mg/mL, respectively, suggesting that these two extracts may present minor toxicity to humans. Three **SB** extracts (SBW, SB30, and SB60) showed potent activities against HIV-1 PR, with respective IC_50_ of 0.006, 0.028 and 0.03 mg/mL. However, no inhibition was observed for any **SB** extract against Cat L PR. SB85 did not show any activity towards the three PRs. These results indicated that polar **SB** extracts could potently inhibit HIV-1 PR.

### 2.2. Identification and Quantitation of Ingredients in ***SB*** Extracts Using UPLC-HRMS

**Figure 1:**
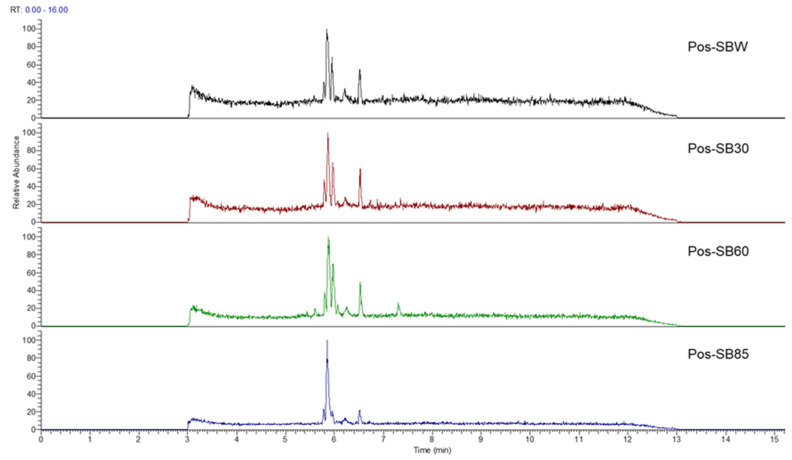
Bioactive flavonoids in **SB** extracts analyzed by UPLC-MS (positive).

**Figure 2:**
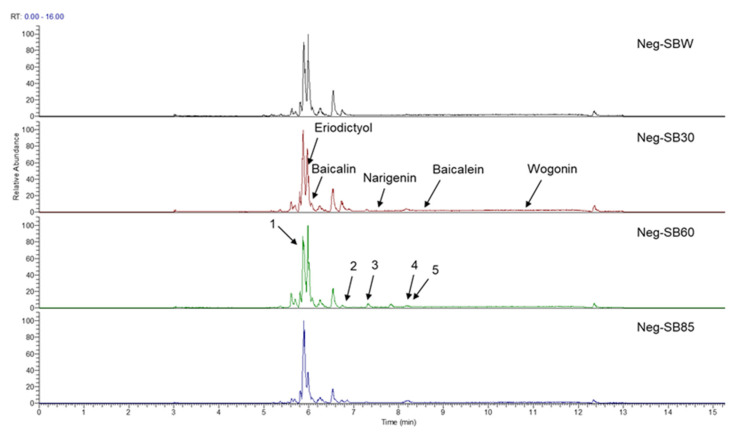
Bioactive flavonoids in **SB** extracts analyzed by UPLC-MS (negative).

In this study, we identified nine compounds inhibiting HIV-1 PR and six compounds inhibiting Cat L PR from **SB**. The five strongest inhibitors (scutellarin, scutellarein, luteolin, hispidulin, and apigenin) were quantitated (Figure 1 and Figure 2 and Table 2). Calibration curves of the five compounds followed a linear regression (R^2^ = 0.9994–0.9998) (Please refer to the Supplementary Materials for details). Scutellarin was the most abundant flavonoid in the **SB** extracts, with contents of 25.18 μg/mg, 23.53 μg/mg, 30.35 μg/mg, and 22.49 μg/mg in SBW, SB30, SB60, and SB85, respectively. The contents of hispidulin and scutellarein ranged from 1.04 to 1.97 μg/mg, except for scutellarein in SB60 (0.74 μg/mg). The contents of luteolin and apigenin were less than 0.3 μg/mg in the **SB** extracts.

### 2.3. Inhibitory Activity of Nine Flavonoids Obtained from ***SB*** against Cat L and HIV-1 PRs, and Their Respective Structure–Activity Relationships

The anti-Cat L and anti-HIV-1 PR activities of the nine flavonoids isolated from **SB** were screened using SensoLyte^®^ 520 Cathepsin L and HIV-1 Assay Kits *Fluorimetric*, respectively, according to a previously reported procedure [20,21]. The nine flavonoid compounds were scutellarin, baicalin, scutellarein, hispidulin, apigenin, luteolin, narigenin, eriodictyol, and wogonin. As shown in Table 3 (Please refer to the Supplementary Materials for details), all nine flavonoids exhibited inhibitory activities against HIV-1 PR (IC_50_ = 0.039–0.83 mg/mL), with luteolin as the most potent inhibitor. Towards Cat L PR, apigenin, hispidulin, scutellarin, scutellarein, eriodictyol, narigenin, and baicalin gave an inhibition of 4.95–37.6% at a concentration of 0.1 mg/mL. However, only the IC_50_ for Scutellarein was able to be determined, at 0.43 mg/mL. We are developing other experimental protocols to confirm the current results and figure out whether there are any behavioral differences between Scutellarein and the other flavonoids in the experimental system. Nevertheless, these flavonoids had a preference for HIV-1 PR and were much weaker inhibitors than the positive controls. 

**Figure 3:**
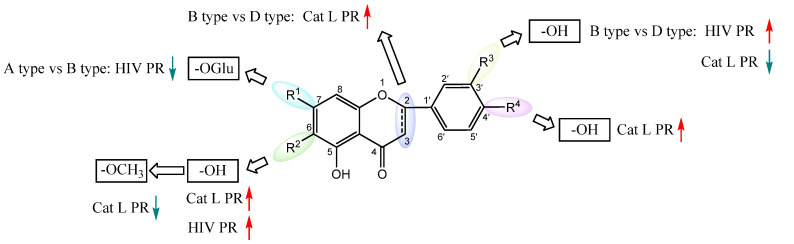
Structure–activity relationships of flavonoids from **SB** against HIV-1 and Cat L PRs.

We further evaluated the flavonoids and activities in their respective categories (Table 3 and Figure 3). For the 5,6,7-trihydroxyl flavone glycosides (A type), scutellarin exhibited a slightly higher activity against HIV-1 PR than baicalin. However, towards Cat L PR, the inhibitory activity of scutellarin was about three-fold stronger than that of baicalin. This implies that 4′-hydroxyl is critical for inhibition of Cat L PR. 

Within the 5,7,4′-trihydroxyl flavone (type B) family, all three compounds, scutellarein, hispidulin, and apigenin, showed potent activities against HIV-1 PR. The stronger activities for scutellarein and hispidulin suggests that C6-subsitution potentiates the inhibitory function. Towards Cat L PR, a moderate inhibition was observed for the compounds. The higher activity of Apigenin implies that C6-substitution is unfavorable for inhibition of Cat L PR. This also illustrates the structural variance and requirements between HIV-1 PR and Cat L PR for binding their respective substrates and inhibitors. Further analysis between type A and type B flavonoids indicated that C7-glycosylation is detrimental to HIV-1 PR inhibition but may be beneficial for Cat L PR inhibition.

For the 5,7,3′,4′-tetrahydroxyl flavones (type C) and 5,7-dihydroxyl-8-methoxy flavones (type E), only one compound was examined in each category. The type C compound Luteolin and type E compound Wogonin showed good inhibition of HIV-1 PR but no inhibition on Cat L PR. Comparison of these two compounds implied that hydroxylation of the C-ring provides better inhibition of HIV-1 PR; however, further studies are needed to evaluate the contribution of C8-methoxylation to HIV-1 PR inhibition. Further comparison of Luteolin with Apigenin suggested that C3′-hydroxylation provides better inhibition of HIV-1 PR for flavone compounds.

In the 5,7,4′-trihydroxyl flavanones (type D), both compounds, Eriodictyol and Narigenin, exhibited comparable effects towards both HIV-1 PR and Cat L PR, although their inhibitions of HIV-1 PR were much stronger. This implies that C3′-hydroxylation has limited effects on PR inhibition for flavanones. Upon comparing these two flavanone compounds with structurally corresponding flavone compounds, Luteolin and Apigenein, we could conclude that the flavone structure with flat conformation of the B-ring is favorable for HIV-1 PR inhibition. However, we could not reach a conclusion about which type of flavonoid (flavones vs. flavanones) is more potent for Cat L PR inhibition.

### 2.4. Molecular Docking Results

Molecular docking was performed for the top four inhibitors (Scutellarein, Hispidulin, Apigenin, and Luteolin) against both HIV-1 PR and Cat L PR [21]. As shown in Table 4, the binding energies of Scutellarein, Hispidulin, Apigenin, and Luteolin were −46.959 to −38.811 kcal/mol towards Cat L PR, and −56.377 to −51.378 kcal/mol towards HIV-1 PR. The docking scores were −8.887 to −6.722 towards HIV-1 PR; however, only Scutellarein showed good docking to Cat L PR (−7.332). These docking results were consistent with the experimental results that the four compounds were potent inhibitors of HIV-1 PR but not Cat L PR.

**Figure 4:**
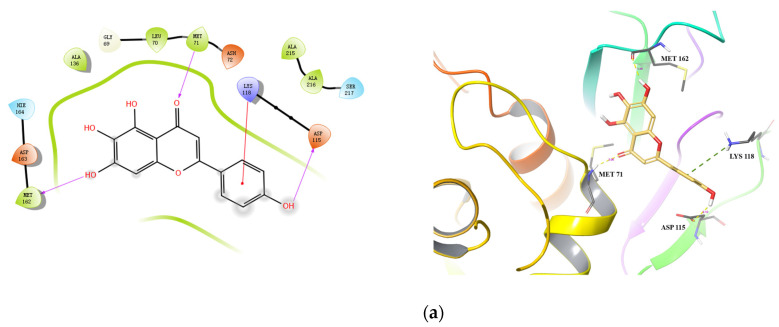

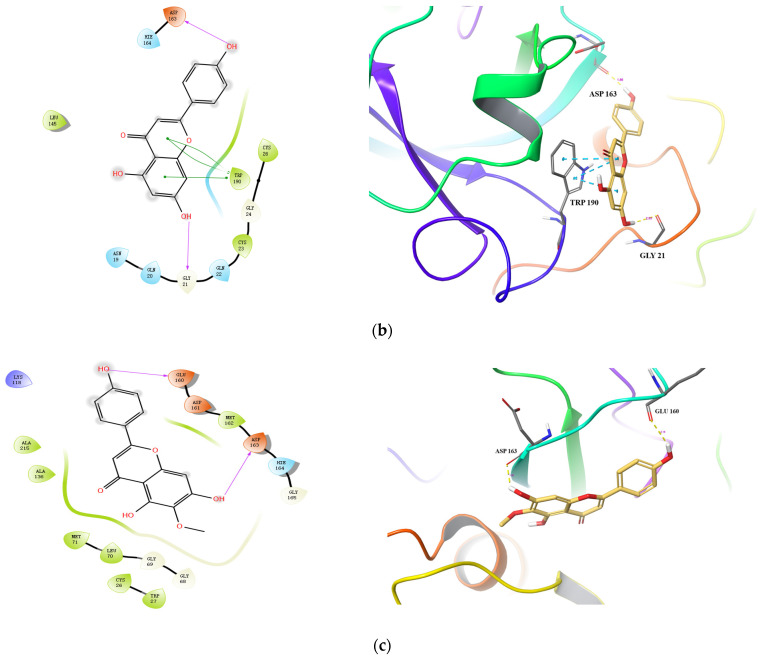
Predicted binding of scutellarein (**a**), apigenin (**b**), and hispidulin (**c**) to Cat L PR. Carbon is colored earth-yellow, while oxygen, hydrogen, nitrogen, and sulfur atoms are colored red, white, blue, and yellow, respectively. The blue dotted lines represent π-π interactions, the green dotted line shows a π-cation interaction and the hydrogen bond is depicted as a yellow dotted line.

Docking conformations of the three compounds (Scutellarein, Apigenin, and Hispidulin) to Cat L PR are shown in Figure 4. Three H-bonds and one salt bridge were formed between Scutellarein and Cat L PR (Figure 4a). The H-bonds were formed between 7-OH and Met162 (1.99 Å), the ketonic functional group at position 4 and Met71 (2.06 Å), and 4′-OH and Asp115 (2.10 Å). For Apigenin (Figure 4b), two H-bonds were formed between 7-OH and Gly21 (2.03 Å) and between 4′-OH and Asp163 (1.86 Å). Three π-π interactions were also formed between A and C rings of Apigenin and Trp190 of Cat L PR. For Hispidulin (Figure 4c), two H-bonds were formed between 7-OH and Asp163 (1.91 Å) and between 4′-OH and Glu160 (2.18 Å). 

**Figure 5:**
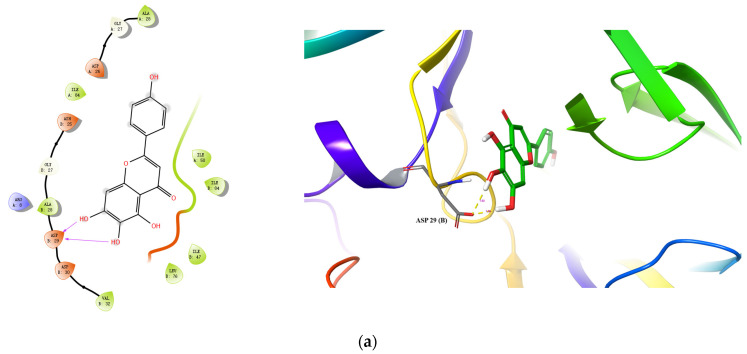

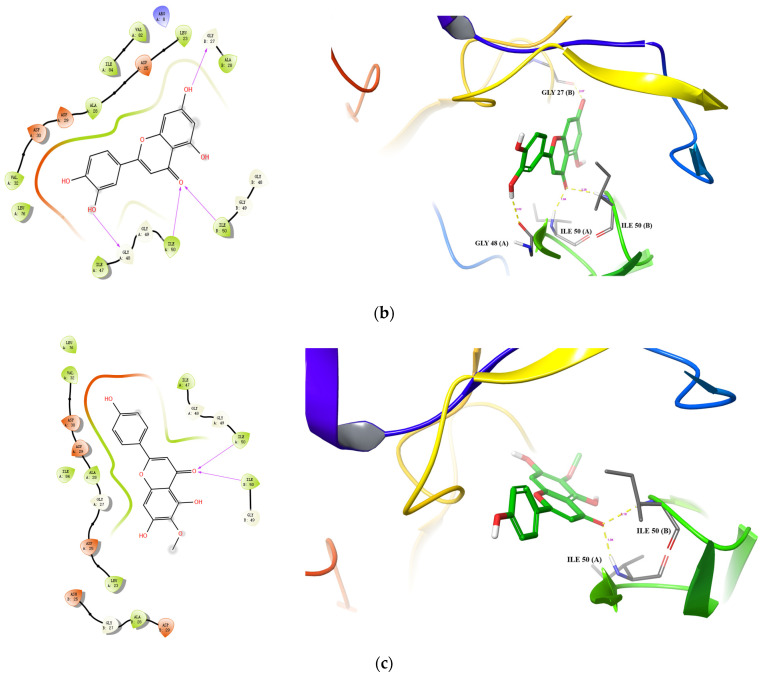
Predicted binding of scutellarein (**a**), luteolin (**b**), and hispidulin (**c**) to HIV-1 PR. Carbon is colored green, while oxygen, hydrogen, nitrogen, and sulfur atoms are colored red, white, blue, and yellow, respectively. The blue dotted lines represent π-π interactions, the green dotted line shows a π-cation interaction and the hydrogen bond is depicted as a yellow dotted line.

Docking conformations of three compounds (Scutellarein, Luteolin, and Hispidulin) to HIIV-1 PR are shown in Figure 5. For Scutellarein (Figure 5a), 6,7-di-OH formed two H-bonds with Asp29(B) (1.66 Å and 1.92 Å). For Luteolin (Figure 5b); 7-OH formed one H-bond with Gly27(B) (2.07 Å); the 4-ketonic functional group formed two H-bonds with Ile50(A) (1.94 Å) and Ile50 (B) (2.28 Å), respectively; and 3′-OH formed one H-bond with Gly48(A) (2.02 Å). For Hispidulin (Figure 5c), the 4-ketonic functional group formed two H-bonds with the Ile50(A) (1.94 Å) and Ile50(B) (2.19 Å), respectively. Taken together, the present docking analysis supports the studies of enzyme inhibition and structure–activity relationships, and may provide modification guidance for the development of more effective PR inhibitors.

## 3. Conclusions

In this study, we determined the inhibitory activities of nine flavonoids and four **SB** extracts against HIV-1 PR and Cat L PR. Three **SB** extracts (SBW, SB30, and SB60) and all nine flavonoids (Luteolin, Hispidulin, Scutellarein, Apigenin, Narigenin, Eriodictyol, Scutellarin, Baicalin, and Wogonin) inhibited HIV-1 PR, with IC_50_ in the range of 0.006 to 0.83 mg/mL. Six flavonoids (Scutellarein, Apigenin, Hispidulin, Narigenin, Eriodictyol and Scutellarin) showed 10–37.6% inhibition of Cat L PR at a concentration of 0.1 mg/mL.

As the most abundant flavonoid in the **SB** extracts, scutellarin exhibited only moderate inhibition of both PRs. Interestingly, as an aglycone and one of the metabolites of scutellarin [22], scutellarein showed potent inhibition of HIV-1 and Cat L PRs. This indicates that free 5,6,7-hydroxyl groups are likely critical for the inhibitions and provide better bonding affinity within the active sites of both PRs. The introduction of 4′-hydroxyl and 6-hydroxyl/methoxy groups was shown to be essential in 5,6,7-trihydroxyl and 5,7,4′-trihydroxyl flavones, respectively, to enhance dual anti-PR activities. Hence, among the 5,6,7,4′-tetrahydroxyl flavones, scutellarein may serve as a leading molecule for developing more effective dual PR inhibitors. The 5,7,3′,4′-tetrahydroxyl flavone luteolin was a selective and potent inhibitor of HIV-1 PR. Quite a few flavonoids, such as quercetin and kaempferol, demonstrated potent inhibitions of HIV-1 PR and deserve further studies to develop more potent PR inhibitors [23,24]. Our current study also revealed that three **SB** extracts (SBW, SB30, and SB60) and their respective active components, including luteolin and scutellarein, exhibited potent inhibitory activities against HIV-1 PR. However, the selected phytochemical samples are poorly soluble in a neutral assay buffer, and this may lower the bio-availability towards Cat L PR. To develop more dual- and even pan-viral protease inhibitors from medicinal plants, further studies are warranted, to explore potential protease inhibitors based on these active components, for the prevention and/or treatment of COVID-19. 

## Figures and Tables

**Table 1:** The IC_50_ values of *SG* extracts against HIV-1, Cat L, and renin PR (*n* = 3).

Name	IC_50_ ± SD (mg/mL)		
	Cat L PR	HIV-1 PR	Renin PR
SGW	>100	0.006 ± 2.79	0.59 ± 4.76
SG30	>100	0.028 ± 6.72	0.70 ± 0.87
SG60	>100	0.03 ± 1.71	>100
SG85	>100	>100	>100
PC1	-	1.7 × 10^−4^ ± 1.91	-
PC2	6.8 × 10^−7^ ± 0.99	-	-
PC3	-	-	9.0 × 10^−4^ ± 0.80

-: no test. PC1: pepstatin A, positive control for HIV-1 protease. PC2: cathepsin L inhibitor, positive control for cathepsin L protease. PC3: positive control for renin protease.

**Table 2:** Contents of the five strongest inhibitors in **SB** extracts.

No.	Rt (min)	Name	SBW	SB30	SB60	SB85	Regression Equation
			Content (μg/mg)				
**1**	5.86	Scutellarin	25.18	23.53	30.35	22.49	Y = 5.76 × 10^3^ X − 8.353 × 10^4^; R^2^ = 0.9996
**2**	6.73	Scutellarein	1.17	1.97	0.74	1.04	Y = 5.05 × 10^3^ X − 6.36 × 10^5^; R^2^ = 0.9994
**3**	7.28	Luteolin	0.04	0.12	0.09	0.14	Y = 9.496 × 10^3^ X + 3.015 × 10^5^; R^2^ = 0.9977
**4**	8.23	Apigenin	0.10	0.15	0.16	0.29	Y = 9.979 × 10^3^ X + 1.689 × 10^5^; R^2^ = 0.9998
**5**	8.32	Hispidulin	1.04	1.72	1.71	1.78	Y = 8.976 × 10^2^ X − 1.397 × 10^5^; R^2^ = 0.9996

**Table 3:** The inhibition of nine flavonoids from **SB** extracts against Cat L and HIV-1 PRs (*n* = 3).

Name	Structures	Cat L PR		HIV-1 PR	
		% Inhibition ± SD at Concentration 0.1 (mg/mL)	IC_50_ ± SD (mg/mL)	% Inhibition ± SD at Concentration 1.0 (mg/mL)	IC_50_ ± SD (mg/mL)
**5,6,7-Trihydroxyl flavonoid glycoside (A type)**					
Scutellarin	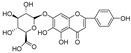	18.3 ± 2.84	>100	56.2 ± 5.30	0.60 ± 6.81
Baicalin	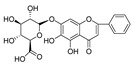	4.95 ± 3.53	>100	53.5 ± 6.45	0.83 ± 6.15
**5,7,4’-Trihydroxyl flavone (B type)**					
Scutellarein	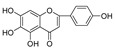	15.6 ± 0.06	0.43 ± 3.17	100.0 ± 0.45	0.068 ± 2.75
Hispidulin	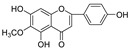	19.8 ± 3.23	>100	86.0 ± 3.76	0.048 ± 2.95
Apigenin	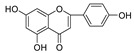	37.6 ± 1.5	>100	91.3 ± 0.87	0.13 ± 3.78
**5,7,3’,4’-Tetrahydroxyl flavone (C type)**					
Luteolin	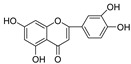	−14.6 ± 10.9	>100	100.0 ± 0.31	0.039 ± 2.42
**5,7,4’-Trihydroxyl flavanone (D type)**					
Narigenin	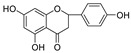	11.2 ± 1.5	>100	89.3 ± 0.00	0.18 ± 1.95
* Eriodictyol	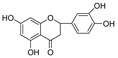	14.3 ± 2.32	>100	100.0 ± 4.98	0.19 ± 5.23
**5,7-dihydroxyl-8-methoxy flavone (E type)**					
Wogonin	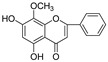	−5.03 ± 2.01	>100	64.8 ± 5.07	0.40 ± 5.29
PC1			4.5 × 10^−7^ ± 2.30		
PC2					1.2 × 10^−4^ ± 1.39

* Reprinted with permission from Ref. [20].

**Table 4:** The docking scores of active flavonoids from **SB** against Cat L and HIV-1 PRs.

Name	Cat L PR (PDB ID:3OF9)			HIV-1 PR (PDB ID: 1QBS)		
	Docking Score	Glide Gscore	Glide Emodel (kcal/mol)	Docking Score	Glide Gscore	Glide Emodel (kcal/mol)
Scutellarein	−7.332	−7.380	−41.662	−6.722	−6.770	−52.931
Luteolin	−3.565	−3.437	−38.811	−8.887	−8.927	−54.263
Hispidulin	−4.172	−4.212	−46.959	−7.543	−7.583	−56.377
Apigenin	−4.919	−4.959	−41.526	−7.220	−7.260	−51.378
Cat L inhibitor	−7.822	−7.823	−93.170	-	-	-
Pepstatin A	-	-	-	−10.940	−10.941	−131.591

“-”: not tested.

## Data Availability

The data presented in this study are available in the article and on request from the corresponding author.

## Supplementary Materials

The following are available online at https://www.mdpi.com/article/10.3390/molecules28114476/s1, 1. The chemical profile of **SB** extracts analyzed by UPLC-MS (positive). 2. Predicted binding models of Cat L PR with scutellarein (4a), apigenin (4b), and hispidulin (4c). 3. Predicted binding models of HIV-1 PR with scutellarein (5a), luteolin (5b), and hispidulin (5c). 4. The quantitative curves and maps of scutellarin, scutellarein, apigenin, luteolin, and hispidulin. 5. Four extracts and the main ingredients of anti-HIV and cathepsin L protease inhibition.

## Sample Availability

The abovementioned samples are available from the authors.
