# Supplementary material for: Identification of Flavonoids from Scutellaria barbata D. Don as Inhibitors of HIV-1 and Cathepsin L Proteases and Their Structure–Activity Relationships
Source: Molecules. 2023 May 31;28(11):4476. doi: 10.3390/molecules28114476 (PMC10254773; doi:10.3390/molecules28114476)
Supplement: Supplementary file 1 [file molecules-28-04476-s001.zip › Qualitative and Quantitative of some compounds.pdf]

Qualitative and Quantitative of flavonoids from *Scutellaria barbata* D. Don Extracts

RT(min)	Name	BW	B30	B60	B80
		Content (µg/mg)			
6.11	Scutellarin	25.18	23.53	30.35	22.49
8.77	Scutellarein	1.17	1.97	0.74	1.04
9.24	Luteolin	0.04	0.12	0.09	0.14
12.51	Apigenin	0.10	0.15	0.16	0.29
12.65	Hispidulin	1.04	1.72	1.71	1.78

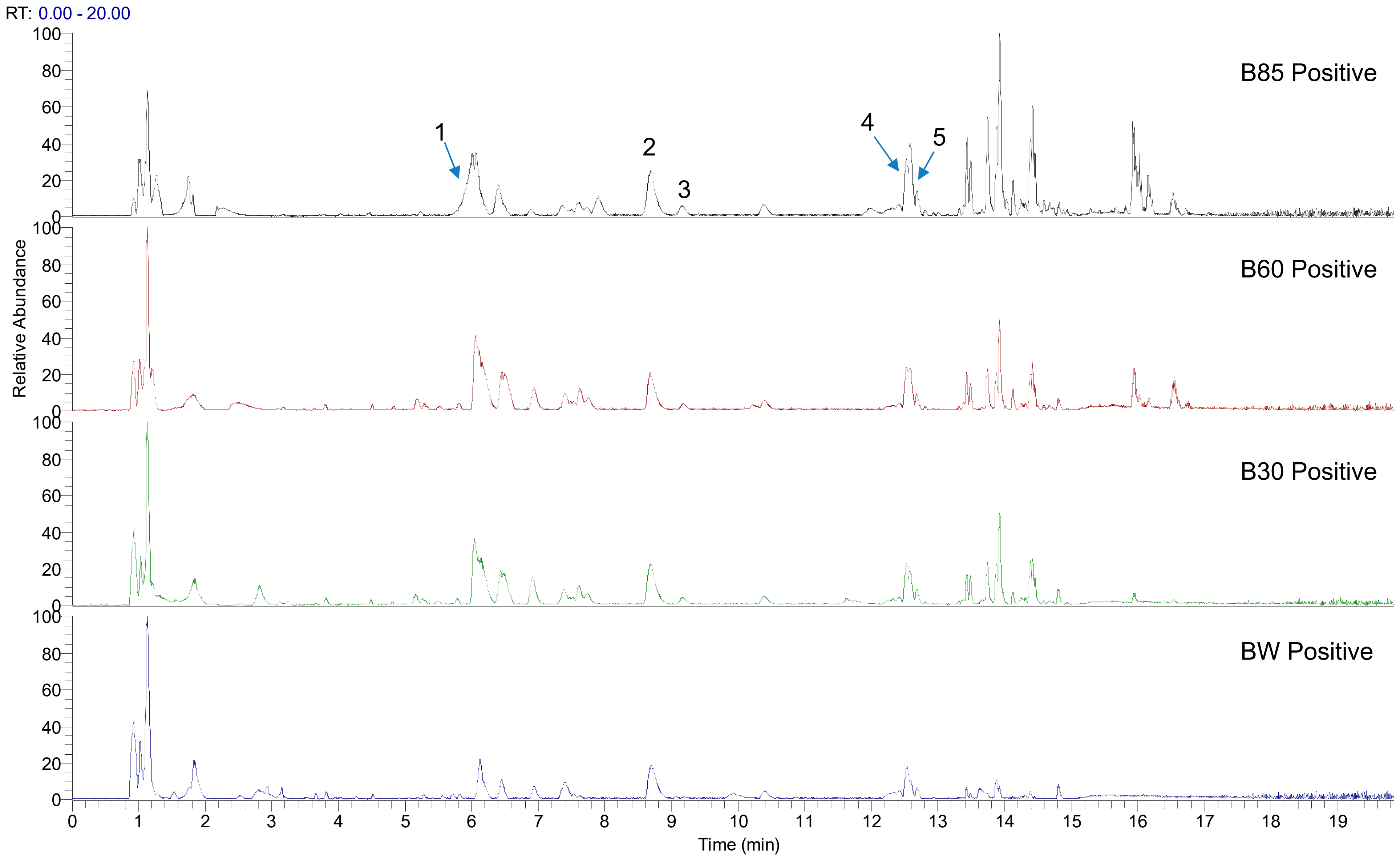

RT: 0.00 - 20.00
B85 Positive
B60 Positive
B30 Positive
BW Positive
1
2
3
4
5
Relative Abundance
Time (min)

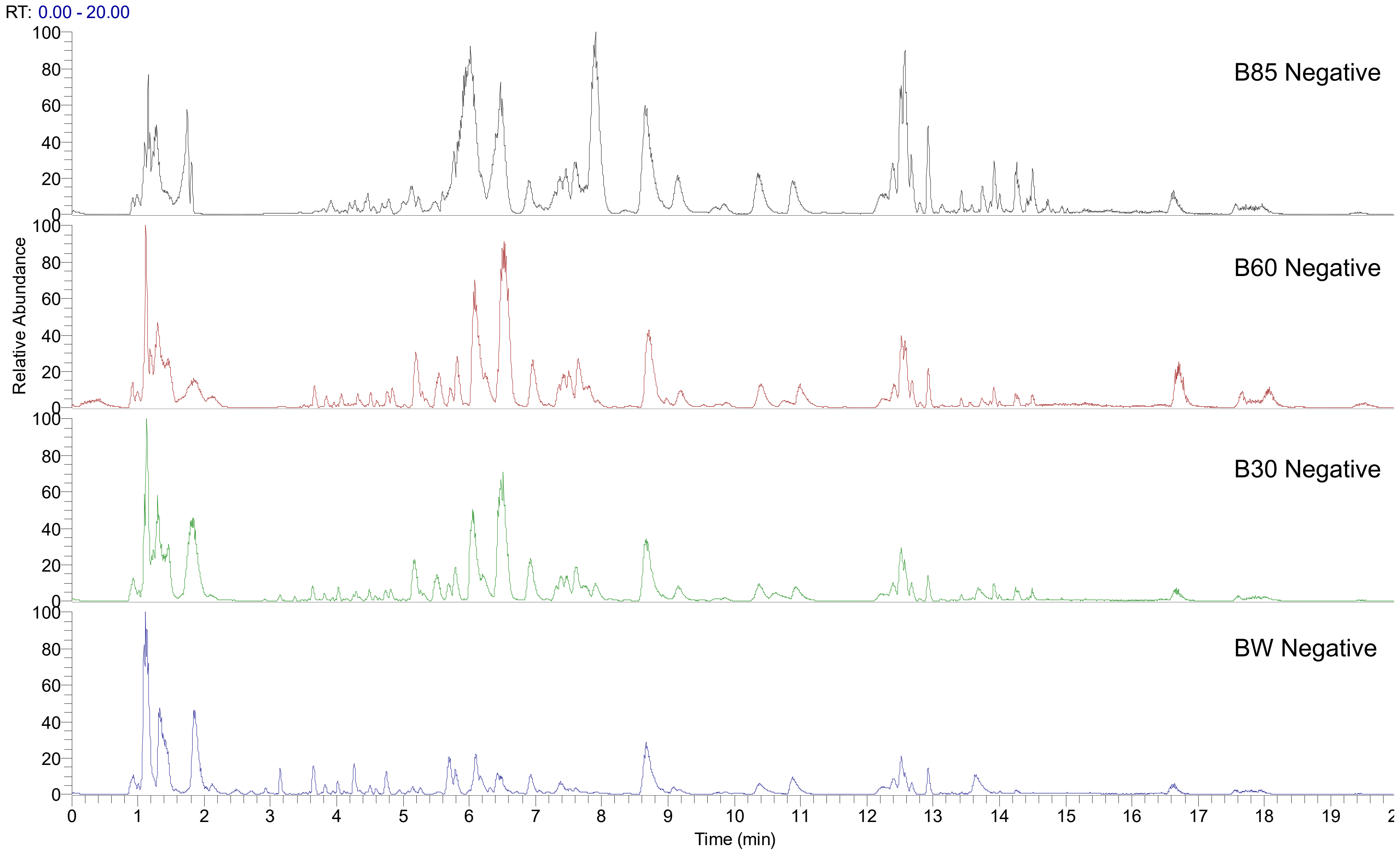

RT: 0.00 - 20.00
B85 Negative
B60 Negative
B30 Negative
BW Negative
Relative Abundance
Time (min)

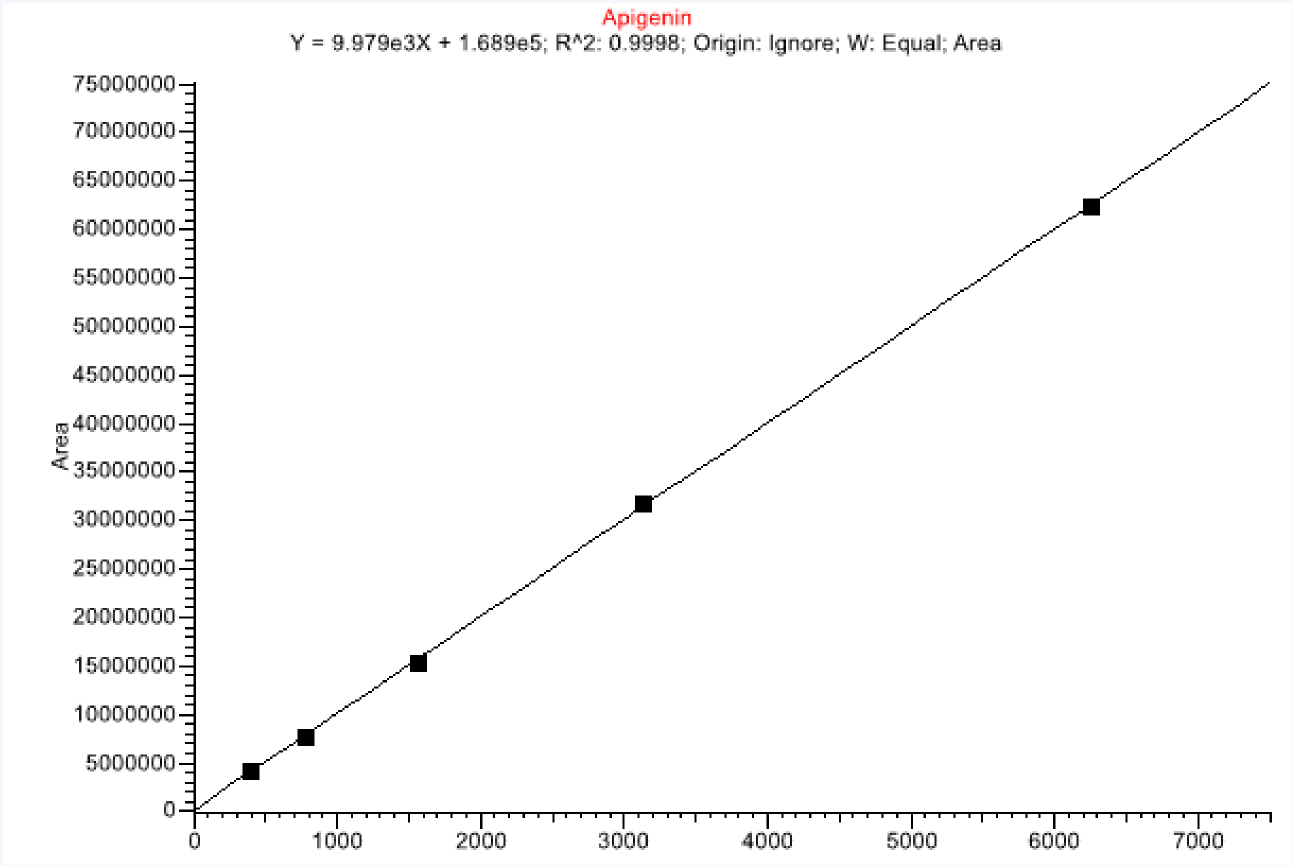

Apigenin
Y = 9.979e3X + 1.689e5; R^2: 0.9998; Origin: Ignore; W: Equal; Area
Area
75000000
70000000
65000000
60000000
55000000
50000000
45000000
40000000
35000000
30000000
25000000
20000000
15000000
10000000
5000000
0
0
1000
2000
3000
4000
5000
6000
7000

Luteolin

Y = 9.496e3X + 3.015e5; R^2: 0.9977; Origin: Ignore; W: Equal; Area

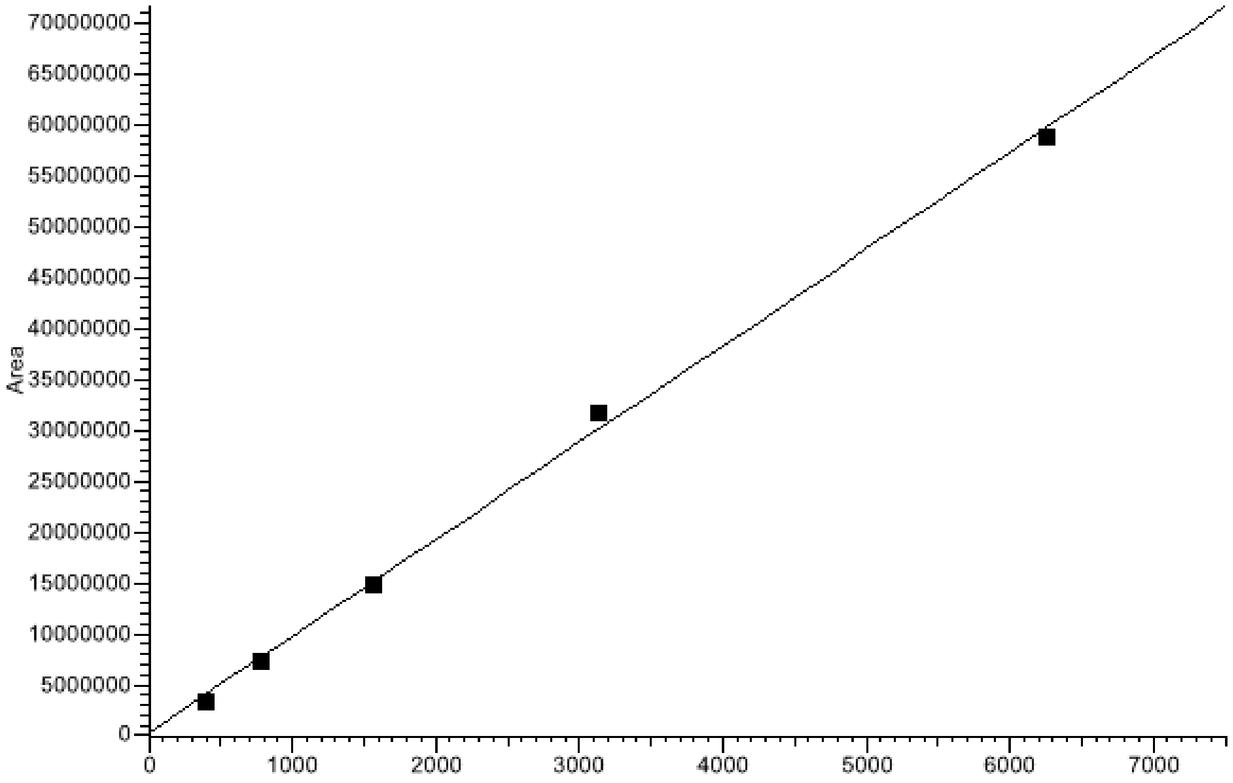

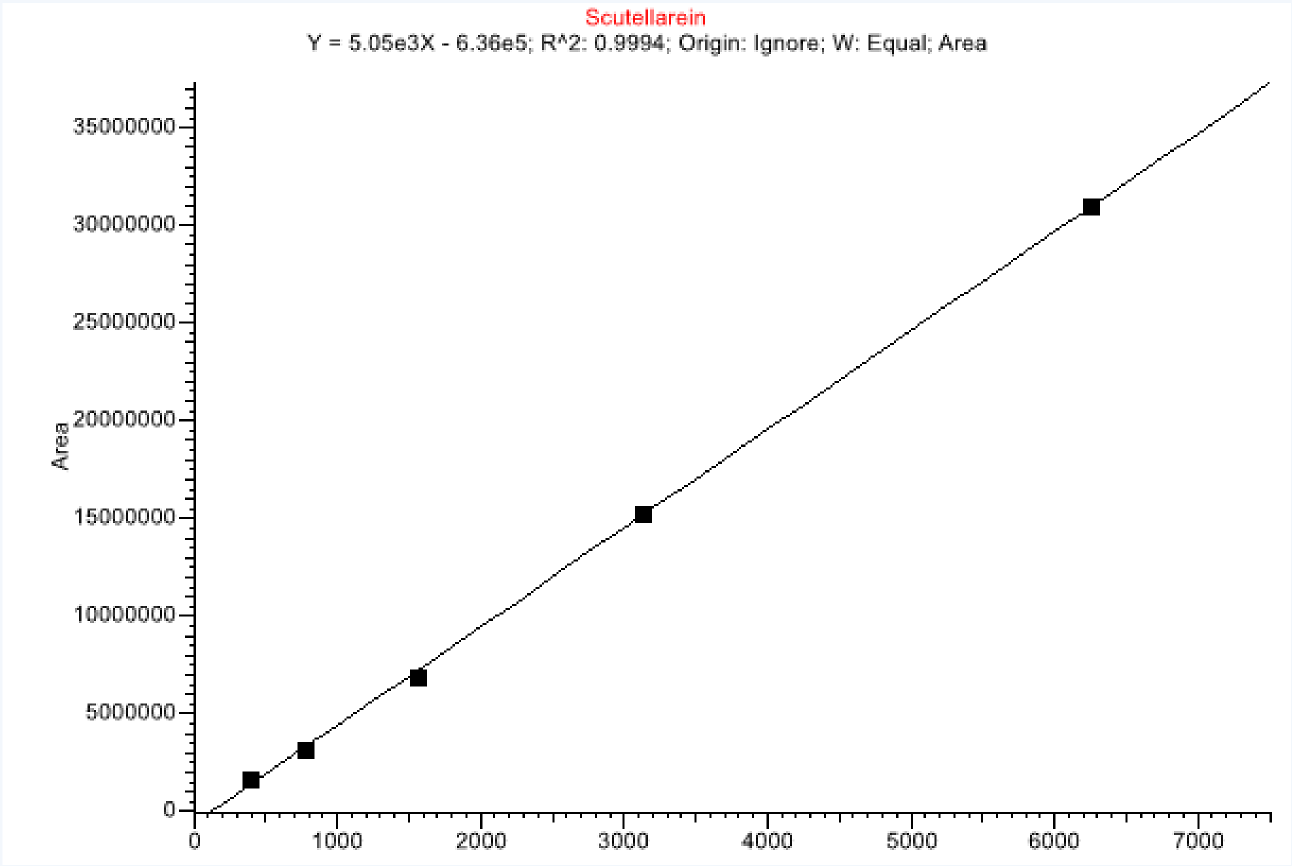
Scutellarein
Y = 5.05e3X - 6.36e5; R^2: 0.9994; Origin: Ignore; W: Equal; Area
Area
35000000
30000000
25000000
20000000
15000000
10000000
5000000
0
0
1000
2000
3000
4000
5000
6000
7000

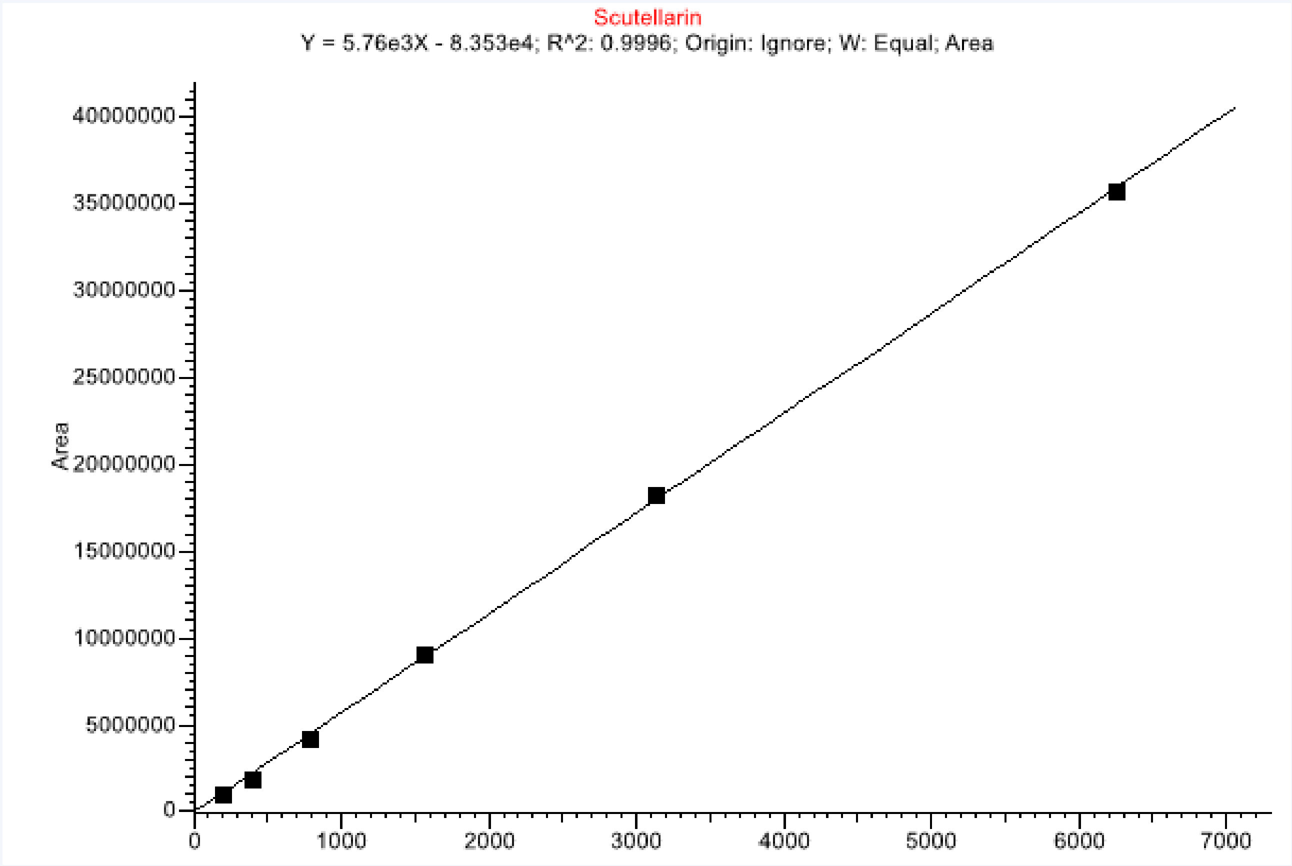
Scutellarin
Y = 5.76e3X - 8.353e4; R^2: 0.9996; Origin: Ignore; W: Equal; Area
Area
40000000
35000000
30000000
25000000
20000000
15000000
10000000
5000000
0
0
1000
2000
3000
4000
5000
6000
7000

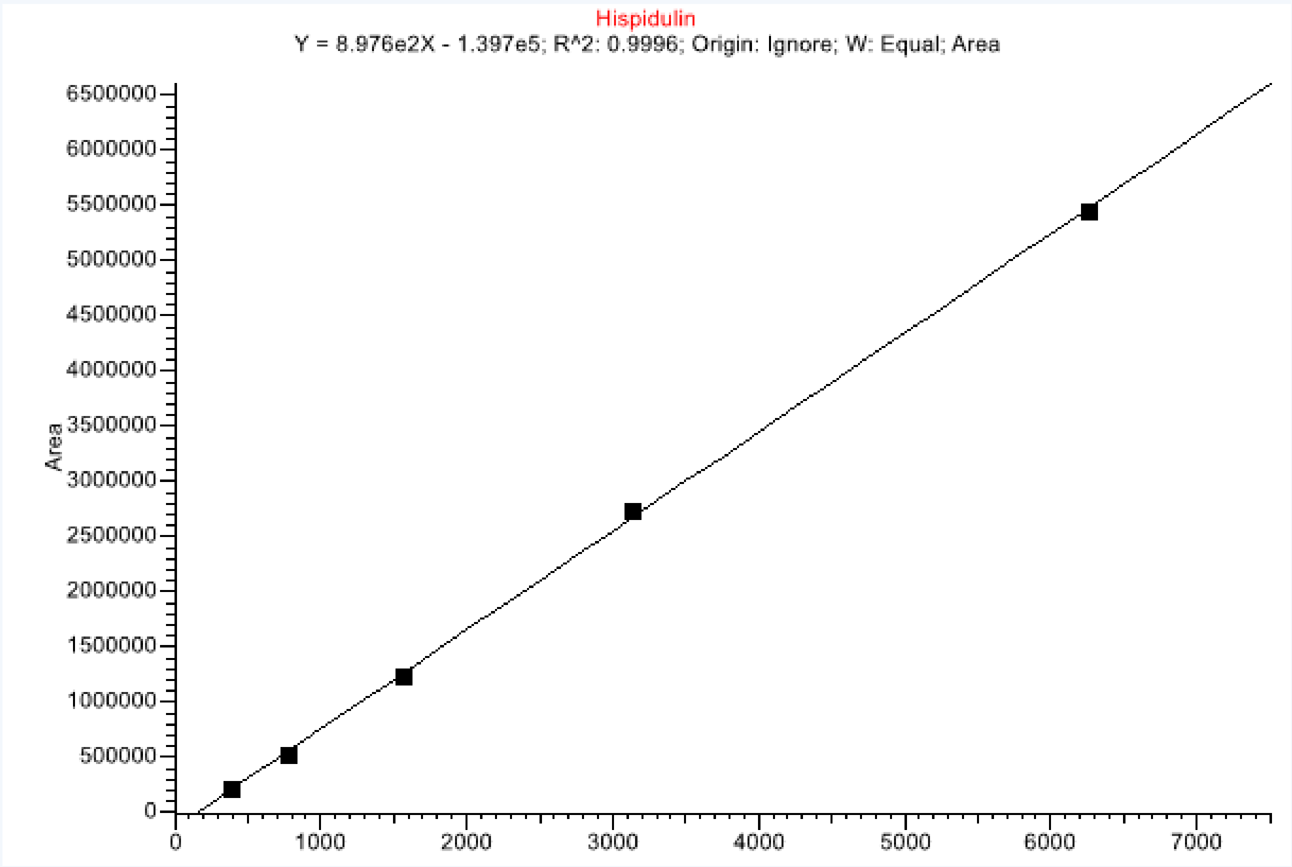
Hispidulin
Y = 8.976e2X - 1.397e5; R^2: 0.9996; Origin: Ignore; W: Equal; Area
Area
6500000
6000000
5500000
5000000
4500000
4000000
3500000
3000000
2500000
2000000
1500000
1000000
500000
0
0
1000
2000
3000
4000
5000
6000
7000
