# Supplementary material for: Identification of Flavonoids from Scutellaria barbata D. Don as Inhibitors of HIV-1 and Cathepsin L Proteases and Their Structure–Activity Relationships
Source: Molecules. 2023 May 31;28(11):4476. doi: 10.3390/molecules28114476 (PMC10254773; doi:10.3390/molecules28114476)

1. Four extracts of *Scutellaria barbata* D. Don against Cat L PR (n=3)

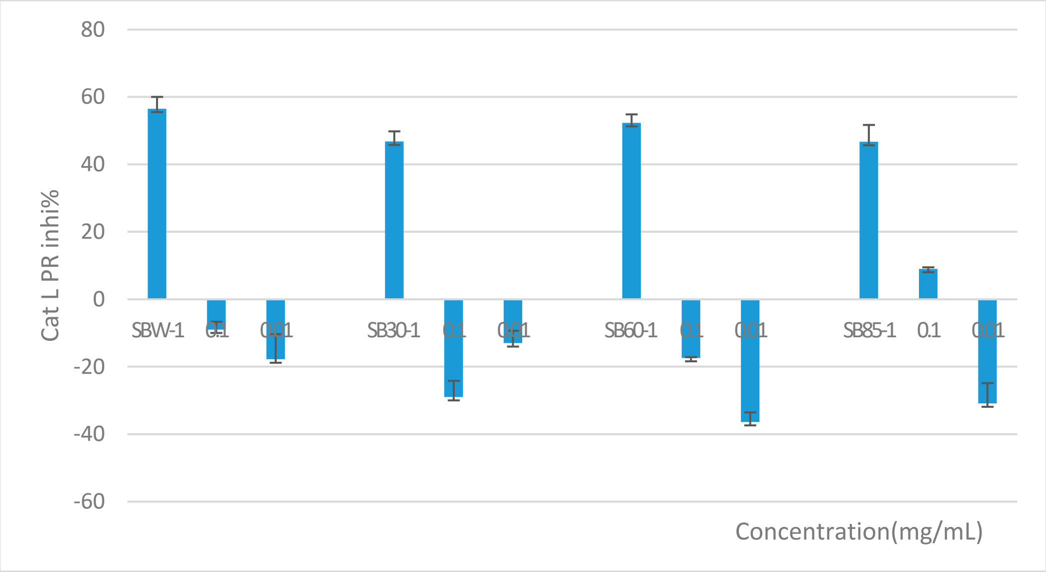

2. Four extracts of *Scutellaria barbata* D. Don against HIV-1 PR (n=3)

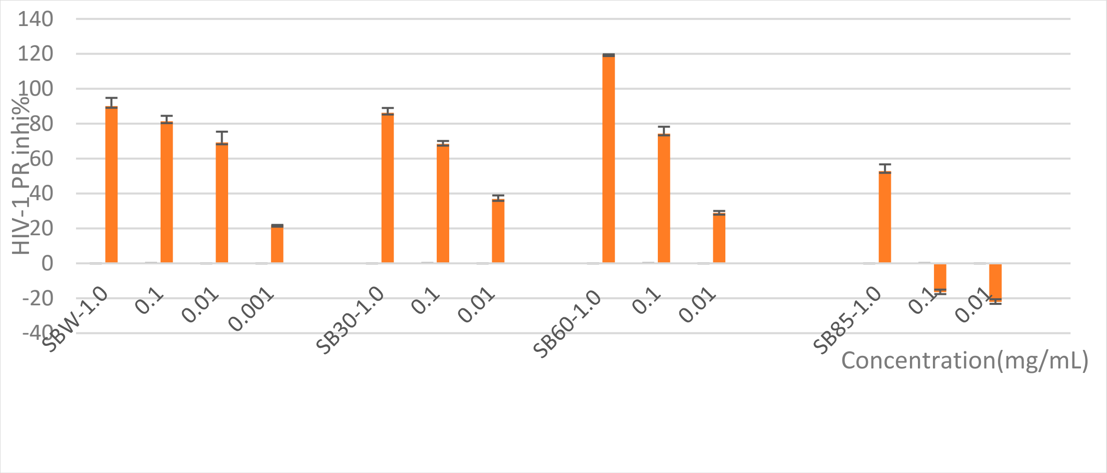

3. Four extracts of *Scutellaria barbata* D. Don against Renin PR (n=3)

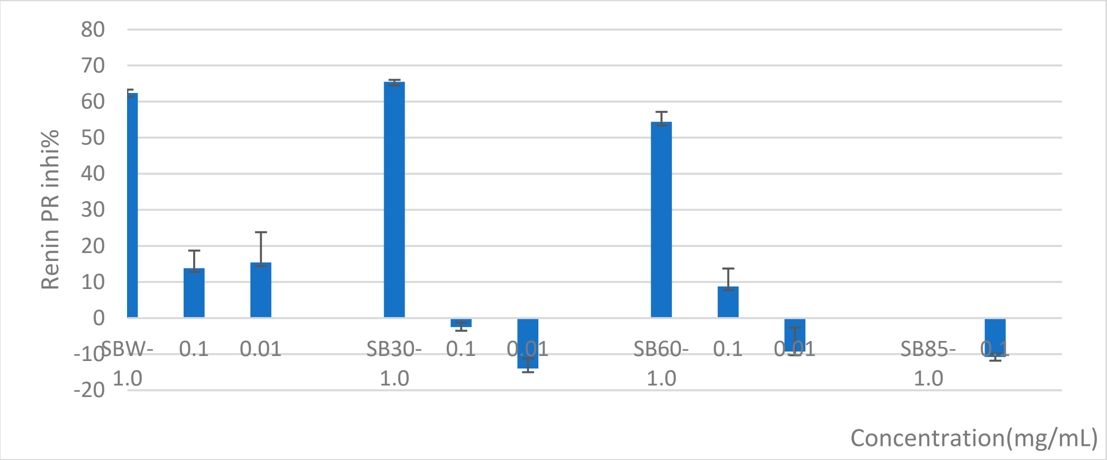

4. Luteolin, Scutellarein and Hispidulin inhibit HIV-1 PR (n=3)

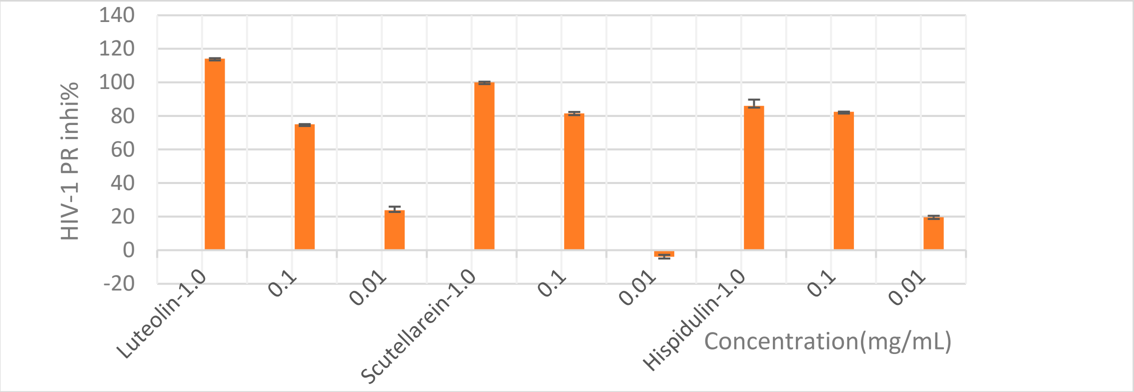

5. Scutellarein，Apigenin and Hispidulin inhibit Cat L PR (n=3)

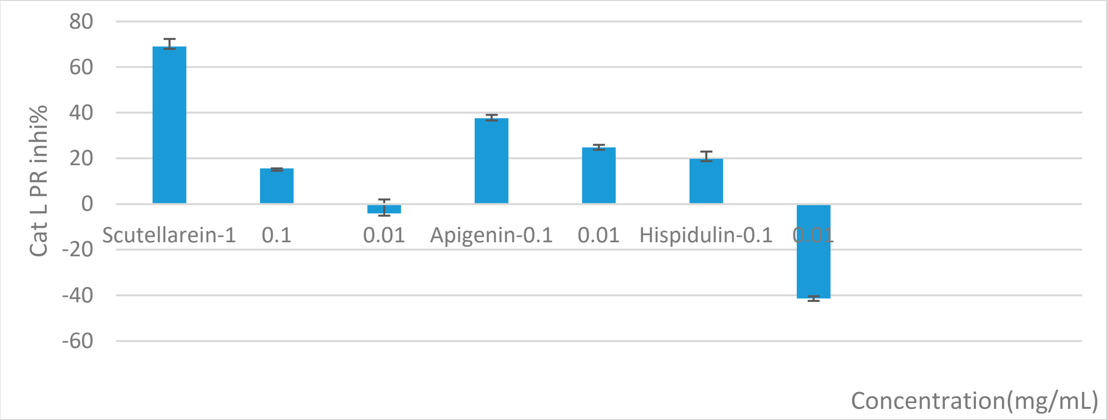

Supplement: Supplementary file 1 [file molecules-28-04476-s001.zip › Test result of SB extracts and main compounds against HIVú1⁄4CatL and Renin PRs.pdf]
